# Automation of dry eye disease quantitative assessment: A review

**DOI:** 10.1111/ceo.14119

**Published:** 2022-06-27

**Authors:** Ikram Brahim, Mathieu Lamard, Anas‐Alexis Benyoussef, Gwenolé Quellec

**Affiliations:** ^1^ Inserm, UMR 1101 Brest France; ^2^ Inserm, UMR 1227 Brest France; ^3^ Université Bretagne Occidentale Brest France; ^4^ Service d'Ophtalmologie CHRU Brest Brest France

**Keywords:** artificial intelligence, automation, dry eye disease, ophthalmology, quantification

## Abstract

Dry eye disease (DED) is a common eye condition worldwide and a primary reason for visits to the ophthalmologist. DED diagnosis is performed through a combination of tests, some of which are unfortunately invasive, non‐reproducible and lack accuracy. The following review describes methods that diagnose and measure the extent of eye dryness, enabling clinicians to quantify its severity. Our aim with this paper is to review classical methods as well as those that incorporate automation. For only four ways of quantifying DED, we take a deeper look into what main elements can benefit from automation and the different ways studies have incorporated it. Like numerous medical fields, Artificial Intelligence (AI) appears to be the path towards quality DED diagnosis. This review categorises diagnostic methods into the following: classical, semi‐automated and promising AI‐based automated methods.

## 1 INTRODUCTION

As developed by the International Dry Eye Workshop (DEWS), dry eye disease (DED) is a multifactorial pathology, affecting tears and ocular surface (Lemp et al. 2007). It is a major pathology, with a growing prevalence due to the increase in longevity, the integration of screen‐based activities at all ages, and environmental pollution; all conditions that induce meibomian dysfunction. Additionally, refractive surgery, whether corneal or intraocular, can cause transient or chronic dry syndrome through an inflammatory and neurogenic mechanism if homeostasis is not restored.^1^ These circumstances of occurrence have been added to the classical etiologies represented by the deficiencies of tear secretion observed in primary or secondary Sjogren's syndrome (SjS) syndrome and the immune or allergic causes, which remain the source of severe dry eyes. DED results in tear film instability, damaged ocular surface and thus in discomfort and pain.^2^ It also decreases visual performance in terms of visual acuity but also in the quality of vision (fluctuations, degradation of contrast sensitivity, optical aberrations induced by the instability of the tear film). The impact on the quality of life can be major and responsible for a significant rate of absenteeism at work and induced depression. This added to the increase of the lubricant market more than 25% in 10 years, placing DED at the rank of public health problem in industrialised countries.^3^

The following review explores these diagnostic tests and the practicality of automating the quantification methods. Each section considers the quantification of one characteristic that signals dry eye. Our goal is to shed light on which diagnostic methods are more than likely to benefit from automation. We also want to show the most prominent studies for each of the diagnostic methods that indicate DED. We believe such a study is of importance to clinicians that are interested in the impact AI has, and will continue to have, specifically in the field of dry eye. This review shows the course of DED diagnosis which, like various fields, is headed towards incorporating more AI solutions. The major goal of our analysis is to emphasise the present demand for automation. The methods described differ in terms of what they quantify, the amount of quantification they accomplish, and the level of automation they employ. However, the overall tendency in our analysis is that automation resulted in either easier acquisition or better accuracy. This review shows the course of DED diagnosis which, like retinal image analysis for instance is headed towards incorporating more AI solutions.^4^, ^5^, ^6^, ^7^, ^8^

The Tear Film Ocular Surface Society (TFOS, DEWS I, DEWS II) presented two reports in 2007 and 2017, which updates the definition and how we classify DED^2^, ^9^. The DEWS II report provides an etiological classification of DED and the basis of its symptoms. DED was divided into two major subtypes: evaporative and aqueous‐deficient. Evaporative DED is due to a high evaporation rate of the tear film, caused intrinsically, meibomian oil insufficiency, or extrinsically. The most common cause of evaporative DED is Meibomian gland dysfunction (MGD).^10^ MGD changes the tear components causing instability and goblet cell loss.^11^ Evaporative DED is often due to extrinsic causes, including vitamin A deficiency, contact lens wear and the use of topical agents.^2^ Aqueous‐deficient DED is due to a decrease in tear production from the lacrimal glands. It can be categorised into SjS related or non‐SjS related. Ten percent of patients diagnosed with DED also have SjS, and both are difficult to diagnose.^12^ Dry eye diseases can be diagnosed by the lack of stability of the tear film, the production of tears, the damage caused to the epithelium or gland dysfunction.

Following both DEWS studies we decided to organise our review into four main sections that each addresses a way of diagnosing DED. Each section contains three subcategories: classical diagnostic methods, semi‐automated and fully automated methods. Section 2 includes methods that evaluate a decrease in tear secretion and volume. Reproducible tests that measure secretion, meniscus shape, regularity and residual volume of tears are key indicators of DED. Section 3 considers methods that focus on the damaged ocular surface. The instillation of a dye penetrates the lipid layer of the epithelium, staining‐damaged areas. The staining can quantify the damage and its severity, but there are limitations of ocular surface damage grading. Following that, Section 4 tackles methods that measure tear film stability. The quality of each layer within the tear film is essential to characterise DED. Section 5 discusses meibomian gland diagnostic methods and examining the lid margin. Glands of the ocular surface produce tear fluid components and diagnosis can be of tear content or glandular structures. This section focuses on MGD diagnostic methods as it significantly correlates with DED. The subsections of each include classical diagnostic methods implement simple medical tests and often require full participation of a specialist. Semi‐automated methods exploit either automated acquisition or algorithms that help in quantifying DED but still require manual intervention. Alternatively, fully automated methods cut out the need for manual intervention and in some cases, automate both the diagnosis and DED severity grading.

Several reviews and surveys also have similar objectives but focus mainly on classical clinical diagnostic methods.^13^, ^14^, ^15^, ^16^, ^17^, ^18^, ^19^, ^20^ Other articles and reviews discussing current and future AI applications in ophthalmology include.^7^, ^8^, ^21^, ^22^, ^23^, ^24^, ^25^, ^26^, ^27^

### 1.1 Method of literature search

The systematic approach for this review included the following search terms: dry eye disease, dry eye, quantification, automated. Using Elsevier's Scopus as a main database, as well as arXiv and IEEE Xplore. We included papers published up to beginning of March 2021 (included). As for the selection process, abstracts of the search results were used to identify methods that can be used. Classical methods were mostly found in clinical papers, as well as existing surveys. As for automated methods or semi‐automated methods, those chosen had to include quantification in their results. The framework should also present clearly where the automation was incorporated.

## 2 TEAR SECRETION AND VOLUME

The reduction in tear secretion is a primary indicator of DED. Tear film is composed of three layers: oil, water and a mucin layer. The aqueous and mucin layers are a single layer of mucoaqueous gel with a viscosity gradient along the tear film.^28^ A deficiency in any layer, ultimately causes discomfort and disrupts the tear film.

### 2.1 Clinical diagnostic tests

Assessing tear secretion dates back to 1903 when Schirmer^29^ first presented his test. The test determines whether enough tears are being produced measuring both basal and reflex tears.^17^ The test uses a small piece of filter paper (35 × 5 mm) placed over the lower eyelid for 5 min. The length of the wetted filter paper is the tear secretion grade. Schirmer describes variations of the test, including using a topical anaesthetic and nasal stimulation to measure reflex tears. Despite the controversy and lack of reproducibility, sensitivity and specificity,^16^, ^30^, ^31^ the test is still frequently used. Moderate modifications have been made to reduce them but without major improvements.^31^, ^32^, ^33^, ^34^, ^35^, ^36^, ^37^, ^38^, ^39^ Further modifications include the fluorescein clearance test, which assesses tear clearance or turnover rate. Fluorescein clearance test consists of 1‐min Schirmer's tests performed consecutively for 30‐min after the application of the fluorescein dye.^40^, ^41^ Tear clearance rate is the rate at which the dye fades 5‐min after instillation and is graded visually: 1–1/256. Dogru et al.^42^ showed that strip meniscometry eliminates the need for fluorescein dye, or any touching of the eyelid and can be performed in 5 s to evaluate tear secretion. Lastly, tear function index value was proposed by Xu et al.,^43^ which consists of both Schirmer's test and measuring the tear clearance rate. Scores correlated with tear quantity and stability and can be computed as follows:

$$\text{Tear function index}=\frac{\text{schirmer test value}}{\text{tear clearance rate}}$$

### 2.2 Semi‐automated methods

All the semi‐automated methods measure the tear meniscus height from images after instilling fluorescein dye. First, used by Guillon in 1998, images were acquired from a video camera attached to a slit‐lamp or an interference device.^44^ Tearscope‐plus (Keeler, Windsor, United Kingdom) was also used to visualise the tear meniscus height by Uchida et al.^45^ in healthy, DED and SjS subjects. The captured images were later analysed using a software to obtain the tear meniscus height. Fodor et al.^46^ compare the measurement of tear meniscus height using images from Keeler Tearscope‐plus (Keeler Instruments Inc.) against slit‐lamp images with or without fluorescein staining. Measurements of the lower tear meniscus height using a tearscope were found to be more repeatable than fluorescein tear film break up time and Schirmer's test. Optical coherence tomography (OCT) is a more recent non‐invasive technique that can scan the tear meniscus in cross‐section. A modern acquisition method is the Topcon ImageNET 2000 (Topcon, Japan), in Johnson et al.’s study,^47^ includes a full program of image enhancements such as; contrast adjustment, box enhancement, are measurement tools, to help the evaluation process. Tear meniscus height measurements were compared between OCT, optical pachymeter and video capture en‐face (IMAGEnet2000), optical pachymetry en‐face and in cross‐section.^47^ The study found all five techniques to have a similar average tear meniscus height. Savini et al.^48^ concluded that OCT can be used to measure tear meniscus height. Raj et al.^49^ also found a positive correlation between Schirmer's and tear meniscus and concluded that OCT enables a better understanding and treatment of DED. Tear meniscus height measurements obtained by frequency domain OCT and a keratograph were compared by Baek et al.^50^ and Arriola et al.^51^ Baek et al. concluded that tear meniscus height measurements correlated with each other and other tests [Schirmer test, tear film breakup time (TBUT), dry eye severity] with good repeatability while Arriola et al. found more reliable measurements using the frequency domain OCT and weak correlation with keratograph.

Courrier et al.^52^ presented an all‐in‐one non‐contact ocular surface imaging device (LacryDiag‐Quantel Medical) that measures various dry eye parameters that include the tear meniscus height. It also provides lipid layer analysis, non‐invasive breakup time (NIBUT), and meibography analysis, which all require manual intervention to obtain the final quantification. Other devices capable of tear meniscus height measurement include Dry Eye Monitor KOWA DR‐1 (Kowa), Oculus Keratograph 5M (K5M; Oculus Optikgerate GmbH) and Optovue RTVue (Optovue Inc.).

### 2.3 Automated methods

Yedidya et al. presented an algorithm that evaluates the tear meniscus from slit‐lamp images after fluorescein instillation.^53^ Based on asymmetric graph‐cuts, the method segments the tear meniscus and determine its grade through height and number of branches. Another method presented by Cheazemin et al.^54^ also automatically measures the tear meniscus height from video sequences. Using k‐means segmentation, and a threshold determined using the Otsu's method, tear meniscus height was determined based on the length of the segmentation. The method requires calibration to obtain a physical value from the measurement unit (pixels). The first two methods operate on the same imaging modality (slit‐lamp images) and are based on similar image processing techniques.

A more recent method that also automatically assesses the characteristics of the tear film including, tear meniscus, area, height, depth and radius was presented by Stegmann et al.^55^ Using ultrahigh‐resolution OCT measurements to automatically segment the tear meniscus and extract the tear film characteristics. It was tested on 10 healthy subjects only, a significant correlation was found between all tear meniscus parameters and literature values. All three methods showed that tear characteristics can be extracted with simple methods and two data types, namely slit‐lamp images and ultrahigh‐resolution OCT, although none were extensively validated.

## 3 OCULAR SURFACE DAMAGE

The lesions that appear on the epithelial and sub‐epithelial tissues are clinical signs of various ocular surface diseases.^56^ Referred to as superficial punctate keratitis (SPK),^57^ which is a small damaged groups of cells on the surface of the cornea or conjunctiva.

### 3.1 Clinical diagnostic tests

The damaged parts of the cornea are made more visible using a dye. Fluorescein dates back to 1882 when it was first used to stain corneal abrasions.^58^ Fluorescein sodium is still used and instilled by preserved doses or paper strips. Lesions are more visible if a yellow (blue‐free) filter is used.^16^ Damage to the conjunctival epithelium however is more difficult to detect with fluorescein staining due to the poor scleral contrast. Another dye, derivative of fluorescein, rose bengal is mainly used on the conjunctiva to detect the damage areas.^16^ Lastly, lissamine green is a synthetic organic acid dye that is interchangeable with RB^15^ and used more often since it's been proven to be less toxic and more easily tolerated. Bron et al. present a more exhaustive coverage of clinical ocular surface staining.^59^ To quantify the staining and track its evolution, clinicians refer to numerous grading scales that were detailed by Begley et al.^13^ Another more detailed method for grading recently developed by Woods et al. includes a scale of 0–100 for staining type and area, and 0–4 for depth.^60^ Named the CORE (Center for Ocular Research Education) staining scale, and is reported for five zones [central (C), superior (S), nasal (N), inferior (I) and temporal (T)]. The staining scale helps in tracking the evolution of the damage.

### 3.2 Semi‐automated methods

The following methods process slit‐lamp recorded images with a semi‐automated algorithm. Ornberg et al.^61^ improve fluorescein images with image processing techniques. The stains are also measured by size, intensity and position relative to the pupil, which improves quantification. The method does not automate the staining quantification but helps in identifying them. Amparo et al. compared their corneal fluorescein staining index, an index given using their proposed method, to the National Eye Institute/Industry grading scale.^62^ The proposed method divides the cornea in five zones and quantify each of them separately. The corneal fluorescein staining index scoring system summarised the result in a 0–100 score. The corneal fluorescein staining index provides a plateau gap that allows for the evolution of the staining to be documented. The corneal fluorescein staining index has a larger grading range and is computer guided, which resulted in increased consistency and accuracy compared to the National Eye Institute/Industry grading scale. Although not fully automated, the method brings forward a great contribution which is a new grading scale. It also has a better interobserver agreement when compared to that of the National Eye Institute/Industry grading scale. Another study using lissamine green dye by Bunya et al. evaluates ocular surface damage only to the bulbar conjunctiva.^63^ The image is then processed and a random forest regression classifier is trained using the extracted features. The method uses automated acquisition and grading but requires user assistance for the region of interest selection. The scores correlated with the van Bijsterveld scale better than the National Eye Institute/Industry grading scale.

### 3.3 Automated methods

Chun et al.^64^ propose a method that takes advantage of several digital image processing steps to enhance corneal punctate epithelial erosions. The method uses median filters, noise removal, contrast‐limited adaptive histogram equalisation filters that improve contrast without amplifying the noise, as well as erosion filters to better highlight the ocular surface damage.

The method resulted in a strong correlation with the Oxford scale and a better correlation with the National Eye Institute/Industry grading scale than both methods by References 62 and 63, which were similar. Another automated method of staining detection that uses image processing was developed by Rodriguez et al.^56^ The binary images are used for blob detection, and those are the punctate dots. The algorithm then counts the dots (*N*_*dots*_). Lastly, the predicted grade (*G*_*pred*_) is calculated using Equation (1).

(1) $$G_{\text{pred}}=1.48\log\left(N_{\text{dots}}\right)-0.206$$

(2) $$G_{\text{pred}}=1.3244\log\left(N_{\text{dots}}\right)-0.0612$$

A similar approach summarised with a different Equation (2) was presented by Bagbaba et al.^65^ The method employs image processing techniques such as Hough transform, active contour models and connected component labelling. This method had a larger data set (70 images) and a better correlation with clinical scores compared to that presented by Rodriguez et al. (54 images). The grades predicted using Equation 2 have a higher precision and thus a better correlation with clinical grading scales.

An approach to quantify staining and distinguish SjS from Ocular Graft versus‐host Disease was recently described by Pellegrini et al.^66^ Pellegrini et al.’s method calculates the corneal staining index, which is defined as the ratio between the detected staining area and the area of the cornea. The particles' morphological patterns are described to help distinguish between SjS and Ocular Graft versus‐host Disease staining. The parameters include area, circularity and roundness of each stain. The study found that Ocular Graft versus‐host Disease staining spots were more circular and round compared to SjS. The approach resulted in a less significant correlation with the oxford and National Eye Institute/Industry grading scales compared with methods by.^56^, ^64^, ^65^ Benefiting from deep learning, Su et al. propose the automatic detection and grading of punctate dots with a convolutional neural network (CNN).^67^ Images were manually segmented only to train the model using five pre‐defined classes: tear film, eyelash, eyelid, punctate dots and conjunctiva. The model then produces a probability map of punctate dots, used to calculate the CNN‐SPK value. A newly defined grading scale, which is:

$$\mathrm{CNN}-\mathrm{SPK}=\frac{\;\text{area of the punctate}\;\mathrm{dot}\;\text{predicted}}{\text{area of the cornea}}$$

Obtaining significant correlations with clinical grading scales, this method is close to those by.^56^, ^64^, ^66^ The more recent fully automated methods all use slit‐lamp examinations and achieve similar results. It is evident that using only staining examinations we can extract valuable information that can help refine ocular surface diagnosis. One advantage of slit lamp acquisitions is that the data, namely images, can easily be extracted and are compatible with popular computer vision AI algorithms. Therefore, we expect significant development along this direction in the near future.

## 4 TEAR FILM STABILITY

A healthy human tear film is composed of a lipid layer, and the aqueous and mucin layer. The evaluation of tear film stability can be through the components or a normal blink function. Any impairment can result in the tear film not reforming properly.

### 4.1 Clinical diagnostic tests

The most referenced and useful technique to assess the extent of tear evaporation is the tear breakup time (TBUT). Introduced by Norn et al. (1969) the test diagnoses the tear film instability by first instilling sodium fluorescein then observing through a slit‐lamp. The timing between the last blink and appearance of the first break or dry spot is TBUT. Results of less than 10 s are abnormal, 5–10 s being marginal, and less than 5 s suggest dry eye.^68^ Vanely et al. also found TBUT to be highly reproducible but stated that it remains a supportive value for DED diagnosis.^69^ TBUT is performed in various ways and is continuously modified. The main difference between the ways is the degree of invasiveness. Some BUT measurement methods instill sodium fluorescein then observe using a cobalt blue light with a yellow filter. Alternatively, non‐invasive methods do not use dye and instead use different instruments. Guillon et al. measured in a non‐invasive manner using a tearscope, giving NIBUT.

Besides TBUT, another way to measure tear film stability is changes in ocular temperature.^70^, ^71^ The temperature change mapped using an ocular thermogram allowed Morgan et al.^72^ to determine that the mean ocular surface temperature was greater in DED patients. Fujishima et al. determined a change in the corneal temperature using an infrared radiation thermometer.^73^ Changes in temperature with each blink were observed to be larger in patients with DED. Both studies showed a correlation between corneal temperature change and TBUT.

Mengher et al. also assessed tear film stability via NIBUT measurements.^74^ The method described is using a slit‐lamp and observing a grid reflection from the tear. Discontinuities in the pattern observed are caused by loss of tear film integrity. The keratometer that measures the corneal curvature and shows an illuminated grid pattern reflected from the tear surface.^19^, ^36^ A modified method using the keratometer, proposed by Hirji et al. includes adding a circular grid and the mean of five measurements to obtain TBUT.^75^ Another instrument used to asses NIBUT is the hand held keratoscope which also uses a grid (Loveridge grid).^76^ Wang et al. underlined the importance of documenting the instrument used when measuring NIBUT.^77^ The comparison study highlighted that instrument‐mounted interferometric and keratoscopic measurements had good repeatability unlike hand‐held device.

### 4.2 Semi‐automated methods

Corneal topography, also referred to as photokeratoscopy or videokeratography is another method used to measure NIBUT.^78^ Their automated acquisition maps the surface curvature of the cornea. Goto et al. implemented a new videokeratography software that captures consecutive corneal surface images every 10 s.^79^ A positive correlation was found between the topographic map and the fluorescein staining TBUT results. Goto et al. also proposed another analysis study of tear lipid thickness through tear interference images that quantify lipid spread time and tear lipid stability.^80^ In this study, interference images are compared with colour charts and the lipid layer thickness is correlated with the intensity of the post‐processed image. There was no quantitative correlation or evaluation for the study.

The degree of irregularity of corneal surface shape can be expressed by the surface asymmetry index and surface regularity index. Surface asymmetry index provides a quantitative measure of the radial symmetry of the four central videokeratoscope mires surrounding the vertex of the cornea and surface regularity index is a measure of central and paracentral corneal irregularity.^81^, ^82^ Tear film stability is measured by timing tear film build up, and changes in surface asymmetry index and surface regularity index. Tear Stability Analysis System was evaluated by Kojima et al.^83^

The system results in 10 consecutive corneal topograms and proved effective. The tear stability regularity and tear surface asymmetry index were derived by Kojima et al. from surface asymmetry index and surface regularity index for analysis. The study resulted in a higher tear stability regularity and tear surface asymmetry indexes for the dry eye group.

Digital videos were used to measure the total area of the breakup and then the TBUT.^84^ Employing only an automated method of acquisition as well as analysis on grey‐scale images, area of the breakup and TBUT were measured using a program that calculates the number of pixels in the area divided by the pixels of the exposed cornea. Chiang et al. studied another non‐invasive diagnosis using an infrared thermal imager system.^85^ DED patients often exhibit a faster cooling of the ocular surface. The study showed that analysing thermal images and ocular temperature decay can be used as a DED diagnostic.

The tearscope records the lipid layer interference pattern and can help assess the tear lipid stability. Rolando et al. used the tearscope as a method of acquisition to count the number of blinks, number of waves of the interference patterns and evaluate changes in their shape and position.^86^ The dynamic lipid layer interference patterns had a very significant correlation with TBUT. Videokeratoscopy also assesses tear film surface quality, the model used by Alonso‐Caneiro et al. relates changes in tear film topography and the placido image obtaining very similar results to Mengher et al.^74^, ^87^ Most recently, the methodology presented by Carpente et al.^88^ includes blink detection and image processing to detect the breakup area. The videos used were recorded using a Topcon DV‐3 camera attached to a slit‐lamp. Frame intensity helps identify blinks and after that the region of interest is located and extracted using image processing techniques. The NIBUT is then detected at the appearance of the break up area.

A study by Niu et al. Comparing three different methods to evaluate TBUT; NIBUT measured by video‐interferometer (DR‐1™), NIBUT by video topography (TNIBUT) and finally using fluorescein dye (FBUT). Results of the study showed that all methods were reliable in tear film breakup evaluation and significant difference was between FBUT and NIBUT, TNIBUT and between NIBUT and TNIBUT. Another by Ali Khan et al. compares BUT and NIBUT in contact lens users and found no significant difference between values.^89^ The study used FBUT, video keratograph and keratometer. Lastly, Vidas et al. found NIBUT measurements by tearscope to have higher sensitivity, specificity and area under the curve.^90^

### 4.3 Automated methods

A fully automated tear film stability diagnosis algorithm is presented by Yedidya et al.^91^ The algorithm analyses videos from slit‐lamp examination (fluorescein instillation) and locates the iris and region of interest. The region of interest in this case are the dry areas where the tear film breaks and appears darker in fluorescent images. The method produces a graph of the evolution of dry areas in real time. An optometrist manually segmented the dry areas for evaluation and the method had a high segmentation accuracy. Shortly after, the method was extended to include TBUT measurement, analysis of tear film thinning, and the detection of meniscus‐induced dryness.^92^ The study reports an average error of 1.06 s for TBUT estimation and detects the region of break with encouraging results (segmentation accuracy ≥84%). Both methods have a very small database and test set but encouraging results.

Another method proposed by Cebreiro et al. also measures the TBUT, by analysing the colour information from tear film videos.^93^ Dark spots characterise the breakup of the tear film, and by extracting the percentage of dark pixels that denotes the frame corresponding to the TBUT. The method also had a small data set but provides a good methodology for automated BUT measurement with results that correlated with experts.

Su et al. proposed two parameters, the temperature difference value and compactness value, which deal with the irregularity of temperature distribution on the tear film.^94^ Results showed statistically significant differences between DED and normal eye group. Compactness value is described as a spatial indicator of tear film stability and both parameters correlate to BUT. The study encourages further development of infrared thermal image systems in order to obtain another non‐invasive DED diagnostic method.

The lipid layer can also be assessed from interferometry images and patterns can be recognised to estimate its thickness. Wu et al. segment interferometry images based on texture to detect film breakup areas.^95^ The automated method is able to classify with a high accuracy. By combining both texture and colour analysis, Ramos et al.^96^ classify interferometry images following the Guillon categories^44^ as well as Wu et al.’s method. Bolon‐Canedo et al. address how automatic classification can be time consuming and proposes a feature selection technique that does not compromise accuracy.^97^ The method correlated well to optometrists' annotations, and achieved a higher classification accuracy than Ramos et al. and Wu et al.

A similar approach by Remeseiro et al. also uses colour and texture analysis on the region of interest to obtain a descriptor.^98^ Interferometry images are categorised based on pattern recognition. The proposed method compares four machine learning algorithms: naive bayes (Finn V. 1996), random tree,^99^ random forest,^100^ support vector machine^101^ and different parameter configurations. With grading scales of five clinical categories, the support vector machine classifier produced the most promising results. Acharya et al. tested the above machine learning algorithms using infrared thermography images along with; the k‐nearest neighbour, decision tree and probabilistic neural network to classify normal and DED cases.^102^ It resulted with a much higher accuracy than Beatriz Remeseiro et al. using probabilistic neural network and k‐nearest neighbour for the left eye and support vector machine for the right eye.^98^

An optimization and expansion on Mendez et al.’s method are proposed by Peteiro‐barrel et al. for lipid layer pattern recognition.^103^, ^104^ The method tested different classifiers: support vector machine, decision tree, naive bayes, Fisher's linear discriminant, and multilayer perceptron to classify tear film. Results showed that class binarization and feature selection both affect the performance of the machine learning algorithms, multilayer perceptron was ranked first regardless of the decision‐making method applied. Several methods have been proposed to classify interferometry images using feature selection and machine learning algorithms.^98^, ^105^ A more advanced study in the incorporation of AI to detect TBUT uses a CNN. Proposed by Su et al.,^106^ digital slit‐lamp recordings were used to train the CNN model. The method first labels patches of each frame into the following: breakup, non‐break, eyelash, eyelid and sclera. The trained model, able to identify patches as break region, then results in a probability map of breakup area. This study is the first CNN application to evaluate TBUT and it resulted in strong correlations with clinical measurements.

DED diagnosis through tear film stability is a very rich form of diagnosis that has seen more automation attempts. There are fully automated devices that are commercial and widely used in healthcare facilities. The LipiView®II Ocular Surface Interferometer (TearScience) is a commercialised system that provides both quantitative assessment and imaging. The system includes blink analysis, visualisation and measurement of lipid layer thickness, and illumination of the meibomian glands. Lastly the NIBUT is automatically quantified by various commercialised systems including Kanghua Ruiming's SLM‐6E(A) Dry Eye Analyser, the LacryDiag, and Antares by Lumenis.

## 5 MEIBOMIAN GLAND DYSFUNCTION

Tear Film and Ocular Surface Society (TFOS) completed a report on MGD in 2011, a leading cause of DED.^107^ The report develops a classification of MGD, assesses the diagnosis, grading of severity and creates a summary of recommendations to further the research. MGD is characterised by terminal duct obstruction and any qualitative or quantitative changes to the glands.^107^ Studies also show the contribution of MGD to ocular surface diseases including DED.^108^

### 5.1 Clinical diagnostic tests

Meibomian glands are responsible for secreting lipid, which makes up the tear film along with an aqueous and mucin layer.^109^ Clinical diagnostic tests commonly visually identify duct obstruction, referred to as diagnostic expression. The examination is performed using slit‐lamp bio‐microscopy to check if the glands are blocked or open. Wang et al.’s study showed that 3 years may be an important time node for DED in primary SjS patients before meibomian glands get affected, signifying the importance of early detection.^110^ The most used method in practice is to apply small physical force to the outer surface of the eyelid and observe the outflow from the glands.^111^, ^112^ Graded using a 0–4 scale, where zero is no obstruction and four is complete obstruction.^113^ Turbidity, a characteristic used in common practice, is also graded 0–4. Zero is for clear meibum and four when it's cloudy and toothpaste like. Bron et al. present a comprehensive classification by grading various features for both lids.^114^

Arita et al. developed and validated various grading scales through the following parameters: telangiectasia, mucocutaneous junction, irregularity, plugging, foaming, thickness, meiboscore and each parameter was scored 0–2 or 0–3.^115^ The six new grading scales proved to be reliable, although the study evaluated only one eye for each subject. These are a few of the classical clinical diagnostic tests, some of which have been enhanced. A detailed study by Foulks et al. includes all the MGD clinical diagnostic methods, classification and grading.^10^

### 5.2 Semi‐automated methods

The most common automated acquisition method is meibography (contact or non‐contact), which is used to visualise the meibomian gland's structure. Two forms are described, a transillumination technique that uses white light first introduced in 1977 by Tapie et al., and infrared meibography where the glands appear as white structures.^116^, ^117^ Robin et al. studied both transillumination biomicroscopy and infrared meibography finding both techniques able to classify clinical MGD.^118^

Devices capable of meibography are Keratograph 5M (Oculus) and the LipiScan Dynamic Meibomian Imager (TearScience, Johnson and Johnson Vision). Pult et al. found better intra and interobserver repeatability when using a five‐grade scale after digital grading versus the four‐grade scale.^119^ Lin et al. also used the Keratogaph 5M and measured the MG perimeter and quantified the tortuosity, an effective MGD index.^120^

Image analysis is one of the main forms of diagnosis in recent studies. The hyperspectral imaging technique, was studied by Shehieb et al. proving that MGD monitoring and early detection are possible.^121^ In vivo confocal microscopy, a non‐invasive imaging technique was used by Randon et al. as a classification method along with the In vivo confocal microscopy score.^122^, ^123^ The study showed a strong correlation between the in vivo confocal microscopy and meibography scores. Qazi et al. and Zhou et al. also use in vivo confocal microscopy.^124^, ^125^

Meibometer is an instrument used for meibomian lipid quantification, although the procedure is invasive, it has been enhanced overtime.^126^, ^127^ Yokoi et al. studied MGD function using both direct meibometry and integrated meibometry and found reduced lipid levels in patients with MGD.^128^ The study concluded that integrated meibometry is less effective but provides a visual of the lipid imprint that could be useful.

Napoli et al. found that both spectral‐domain OCT and infrared meibography have a close agreement when used to evaluate gland changes.^129^ A semi‐automated software by Shehzad et al. used infrared images for analysis.^130^ The software proved to be highly practical and reproducible compared with manually analysed meibography images. Images used in their study were taken using the CSO Sirius Topographer (CSO, Italy), and a correlation was found between manual analysis and the semi‐automated software (used by various operators). Some issues include eyelid folds, focus problems and the software sometimes detects white areas or reflections as MG, which are areas that could be improved. Lastly, and more recently Garcia‐Marques et al. also assess MG using infrared meibography (Keratograph 5M).^131^ With a new metric based on MG visibility, the proposed method measures MGD in an objective and repeatable manner that could aid diagnosis and treatment follow‐ups. A detailed review by Villani et al. looks into the most recent publications on MGD imaging diagnostic techniques with well‐reported studies on humans.^132^

### 5.3 Automated methods

Most of the following fully automated image‐based solutions rely on certain tasks that have been enhanced by AI. Automatic segmentation has been significantly enhanced by deep learning and used to quantify MG dropout. Celik et al.’s algorithm segmented enhanced infrared images and, using an support vector machine, classified them as healthy, intermediate or unhealthy with a good accuracy.^133^ Arita et al. developed a software, which objectively evaluated the MG area for non‐invasive meibography.^134^ It first detects the area, uses image‐processing techniques and then measures it. Some limitations include the need for manual corrections and image quality that could affect the detection of certain areas. MGD can also be detected via tear film lipid layer thickness. Hwang et al. developed an algorithm that can determine the iris radius, locate, track a region of interest, process the lipid layer interference pattern and analyse the lipid layer thickness distributions.^135^ The algorithm obtained results consistent with an ophthalmologist's evaluation. Wang et al.’s proposed model segments and computes atrophy percentage achieving a very good accuracy for both tasks.^136^

Images from the Keratograph 5M and a hand‐held camera were used by Prabhu et al. to train a segmentation model.^137^ Images were processed then segmented and five metrics were measured: gland‐drop, tortuosity, width, length and total number of glands. The proposed method proved to be quite accurate as the metrics derived were close to the ground truth metrics. Also using images from a Keratograph 5M Xiao et al.’s automated method relies on image processing techniques to quantify MGD using newly defined diameter deformation index, gland tortuosity index and glands signal index.^138^ Maruoka et al. evaluated deep learning models and their ability to detect obstructive MGD using in vivo laser confocal microscopy images.^139^ The proposed model detects healthy MG and obstructed MG with a high sensitivity and specificity. Wang et al.’s proposed method also segments MGs, analyzes morphological features and predicts ghost glands.^140^ The paper shows that detecting ghost glands is more challenging than detecting MG, obtaining average scores for segmentation and ghost gland prediction accuracy. Dai et al. enhanced a well‐known segmentation model (U‐Net) to segment MGs as well, achieving a high score in segmentation evaluation metric (intersection‐over‐union) and a repeatability of 100%.^141^ The performance results for segmentation of the gland and MGs correlated well with a manual approach. Another image‐based analysis method quantifies MGD automatically using deep learning by training a model to define and measure contours of each MG.^142^ Khan et al.’s method out‐performed the state‐of‐the‐art methods for detection and analysis of MGD drop‐out area compared to manual segmentation. The LipiView® is also currently used in practice for MGD assessment and imaging. It includes dynamic meibomian imaging and near infrared surface imaging. The system also facilitates patient data management but not disease evolution. MGD quantification is currently seeing a lot of novel developments as the image segmentation task is currently very popular in AI.

## 6 DISCUSSION AND CONCLUSIONS

The ocular surface is a recent concept and represents an anatomical crossroads in which the cornea and tear film play a major role. A subject of great importance, its exploration is becoming more systematic. Particularly in the context of refractive surgery, and Sjogren syndrome (SjS). In routine practice and during clinical trials, quantification is subjective since it's based on a rough estimate.

Effective DED treatment is based on effective quantification. Highlighting the importance of having an objective, reproducible and reliable measurement. As discussed in this, review DED can be detected, measured and assessed in several ways. As a result, there is currently a bias in the evaluation of treatments. A bias stemming from experience, quantification method or even the time taken to evaluate a patient. Based on this and the pathophysiology clarified by the work accomplished in the DEWS, measurement tools have been developed to better quantify the changes of the tear film and its repercussions on the ocular surface.

The objective of developing artificial intelligence (AI) into this field is to obtain a more precise evaluation index, whether simple or composite. Although the standardisation of data for AI training could be time‐consuming and require qualified operators, using the trained algorithm is fast and requires very few resources. Any ophthalmologist can benefit from it, regardless of their specialisation. Making quantification less ambiguous can ultimately help ophthalmologists see patients at any stage of DED and still have a detailed non‐biased accurate history. Allowing for timely detection and personalised treatment. The developments possible are at several levels: improving the grading proposed by the platforms, objectively quantifying the qualitative measurements from the slit‐lamp examination with dyes. This approach is by far the most important, since these indices are the reference examinations for clinical research. The third axis of development would be the multi‐modal approach, gathering all the examinations and parameters to define a severity and/or evolution and prognosis score.

Methods that have incorporated deep learning to evaluate and quantify DED emerged in the last couple of years. Most of the semi and fully automated methods that we presented focus on evaluating tear film stability. Including automation has proved to enhance test repeatability. Full automation, although less, is recent and is growing exponentially. The automated methods include only nine deep learning methods, the earliest being published in 2018. This shows that the improvement to be obtained from deep learning is yet to be solidified. The recent development of imaging tools dedicated to the ocular surface now provides a more precise acquisition and visualisation. In fact, all the new parameters proposed by these new tools remain to be optimised in the definition of the thresholds of normality and in their reproducibility, then to aim at a multi‐modal analysis. An automated method that incorporates various diagnostics can help evaluate treatments, assess how well patients are responding to them across time, also improve clinical trials and standardise them. In our opinion, the evolution of eye dryness can only be obtained through patient‐specific diagnosis, that captures every fine change. It is evident that the severity of DED cannot be accurately detected by a single technique.

Our review's main objective is to make the current strive for automation more evident. We believe it to be most beneficial to clinicians with an interest in AI and deep learning. The presented methods differ in what they quantify, the level of quantification they achieve, and the automation level incorporated. Yet, the common trend shown in this review is that both facilitated acquisition and increased accuracy were gained through automation. We also noted that meibomian gland dysfunction could be the method of quantification that AI can drive forward. MGD examinations have data acquisition method that are easily acquired, just as ocular surface examinations. Slit‐lamp videos and imaging can be easily used in deep learning implementation for automation. A pivotal point in DED research is currently occurring, and vast improvements are yet to be seen.

## FUNDING INFORMATION

This research was supported by funding from the Innovative Medicines Initiative 2 Joint Undertaking (JU) under grant agreement No 806975. The JU receives support from the European Union's Horizon 2020 research and innovation program and EFPIA. It is also funded in part by The Brittany Region through the ARED doctoral program.

## CONFLICT OF INTEREST

The authors declare no conflicts of interest.
